# Characterization of near death experiences using text mining analyses: A preliminary study

**DOI:** 10.1371/journal.pone.0227402

**Published:** 2020-01-30

**Authors:** Vanessa Charland-Verville, Demetrius Ribeiro de Paula, Charlotte Martial, Helena Cassol, Georgios Antonopoulos, Blaine Alexander Chronik, Andrea Soddu, Steven Laureys

**Affiliations:** 1 GIGA Consciousness, Coma Science Group, University and University Hospital of Liège, Liège, Belgium; 2 Brain and Mind Institute, University of Western Ontario, London, Ontario, Canada; 3 Department of Physics and Astronomy, Western University, London, Ontario, Canada; University of Sao Paulo, BRAZIL

## Abstract

The notion that death represents a passing to an afterlife, where we are reunited with loved ones and live eternally in a utopian paradise, is common in the reports of people who have encountered a “Near-Death Experience” (NDE). NDEs are thoroughly portrayed by the media but empirical studies are rather recent. The definition of the phenomenon as well as the identification of NDE experiencers is still a matter of debate. To date, NDEs’ identification and description in studies have mostly derived from answered items in questionnaires. However, questionnaires’ content could be restricting and subject to personal interpretation. We believe that in addition to their use, user-independent statistical text examination of freely expressed NDEs narratives is of prior importance to help capture the phenomenology of such a subjective and complex phenomenon. Towards that aim, we included 158 participants with a firsthand retrospective narrative of their self-reported NDE that we analyzed using an automated text-mining method. The output revealed the top words expressed by experiencers. In a second step, a hierarchical clustering analysis was conducted to visualize the relationships between these words. It revealed three main clusters of features: visual perceptions, emotions and spatial components. We believe the user-independent and data-driven text mining approach used in this study is promising by contributing to the building a rigorous description and definition of NDEs.

## Introduction

Near-Death Experiences (NDEs) are increasingly being reported as a clearly identifiable physiological and psychological reality of clinical and scientific significance [1]. NDEs can be defined as a set of mental events including highly emotional, self-related, mystical and spiritual aspects occurring in an altered state of consciousness classically in the context of a life-threatening condition (e.g., cardiac arrest, trauma, perioperative complications, near-drowning or asphyxia, electrocution, attempted suicide) [2–4]. Moreover, NDEs are classically associated with positive emotions like peacefulness, well-being, happiness and joy. To date, few NDEs reports containing negative emotions have been documented [5,6].

The definition and causes as well as the identification of NDErs (i.e., people reporting NDEs) is still a matter of debate. The phenomenon has been thoroughly portrayed by the media but the science of NDEs is rather recent and still lacking rigorous experimental data and reproducible controlled experiments. It seems that the most appropriate theories to explain the onset of a NDE tend to integrate both psychological and neurobiological mechanisms. The paradoxical dissociation between the richness and intensity of the memory, probably occurring during a moment of brain dysfunction, offers a unique opportunity to better understand the neural correlates of consciousness [7].

Since the popularization of the phenomenon in the late ‘70s, NDEs’ identification and description have mostly derived from questionnaires- and especially from the Greyson NDE scale [8]. The scale was initially constructed by selecting 80 features from the existing NDE literature and subsequently reduced the number of features to a final validated [8,9] 16-item multiple-choice tool used to quantify the intensity of the NDE (i.e., total score ranging from 0 to 32) and to assess core content components of 16 NDE features (see S1 Table). For each item, the scores are arranged on an ordinal scale ranging from 0 to 2 (i.e., 0 = “not present”, 1 = “mildly or ambiguously present,” and 2 = “definitively present”; [8,9]). According to the scale, an individual with a total score of 7 or higher on the scale’s maximum score of 32 qualifies as a NDE experiencer [8]. Using this scale, investigations on NDEs could identify the top reported features of the questionnaire: feeling of peacefulness, out-of-body-experiences, seeing a bright light, alerted time perception [4,10–16].

Although the use of standardized questionnaires is crucial to the empirical investigation of such a subjective phenomenon, their use alone could have the inconvenience of offering restricted choices for assessing the experience. To complement their target, research on NDE now aims to explore the output of rigorous analyses of the raw material, that is, NDE first-hand narratives. To date, only a handful of investigations have aimed to assess the content of NDE reports via text analyses. Most of them have been using qualitative methods. For instance, Hou et al. (2013) assessed interview transcripts using idiographic approach in post-traumatic brain injured patients. It consisted in one researcher analyzing in detail one interview transcript before analyzing the whole sample as compared to what was highlighted in the first one. Their findings revealed four main themes: vision of light, intense feelings of well-being astonishment, and fear; sense of helplessness; supernatural aspect of the experience [16]. Recently, Martial et al. [17] used qualitative text analyses to explore the potential chronology of NDE features in self-reported written narratives. They extracted the most frequent reported sequence: out-of-body experience, followed by experiencing a tunnel, seeing a bright light, and ending with a feeling of peace. However, they observed that the sequence was encountered in a very limited number of NDErs suggesting that no “universal” pattern seemed to be identifiable in their sample [17]. In addition, using qualitative thematic analysis, Cassol et al. [18] could identify a total of 11 major themes in NDE written reports. They included “time-bounded” themes (i.e., themes that have a more limited time duration and generally described as a clear isolated event) like light, return, meeting/encounter, description of scenes, darkness and out-of-body experience; and an altered sense of time as a “transversal” theme (i.e., a theme discussed retrospectively when the individual reflects on their NDE) [18]. In addition to the qualitative approach and also to eventually prevent for the potential biases inherent to human observations and potential prior knowledge of the investigators on NDEs, automated quantitative text analyses are also performed. Using a latent semantic analysis quantitative paradigm (i.e., consisting of determining the semantic similarities across narratives), Lange, Greyson & Houran [19] assessed the content of NDE accounts aiming to investigate on the narratives’ structure and possible hierarchy of the reported features. Their findings revealed that the NDE accounts are highly structured and included seven major clusters. The majority of the clusters referred to transcendent or paranormal themes (e.g., angel, god, church, float, voice) while the other clusters referred to physiological or environmental elements (e.g., pain, bed, shoulder, comfortable, door). They also demonstrated that the intensity the NDEs (based on the Greyson NDE scale total score) could be predicted from the written narratives [19]. Latent semantic analysis has also been used to assess semantic similarity between NDE accounts and those of drug-induced altered states of consciousness, indicating that that the N-methyl-D-aspartate (NMDA) receptor antagonist ketamine resulted in reports most similar to those of NDEs [20]. In our view, automated quantitative text analysis techniques are very promising in our aim to investigate such a subjective phenomenon. Therefore, in this preliminary work, we propose the text mining analysis, that has the added values of being open source, user-independent and data-driven, thus free from predetermined concepts. Text mining is the process of combing through countless pages of plain-language digitized text to find useful information that’s been hiding in plain sight [21]. Text mining offers the opportunity of finding associations and expression patterns in narratives that key word searches and other algorithmic forms would not identify [22]. The technique also permits to cluster and visualize in 2D the statistical association’s strengths between words. Since by definition, first-hand experiences like a NDE are highly subjective material, the structure of the account and the diversity of words used may cause difficulties in extracting what is important in a standardized manner and terminology [23]. Unlike classical quantitative analysis, text mining is used to extract useful information from a text collection via the identification and exploration of patterns among unstructured narratives [21,22]. In this preliminary attempt to use purely data-driven technique on freely expressed written NDEs, we aim to (1) explore the main features (i.e. words elements) reported by experiencers and compare them to the existing literature and, (2) extract a map emerging from the spatial correlation or concurrently appearance among the most reported elements.

## Materials & methods

Participants were recruited via the Coma Science Group’s (GIGA Consciousness, University of Liège, Belgium) website, publications and appearances in media. After taking contact with the research team, the participants were mailed the survey document. It consisted of three parts: first, a demographic questionnaire (age at NDE, gender, time since NDE and clinical data (time since NDE, the medical context in which the NDE took place etiology of coma (anoxic/traumatic/other/no medical cause) and the presence of a life threatening event (i.e. acute coma; a period of unconsciousness >1h; [24]) or not (i.e. medical cause or no medical cause without a coma); second a standardized identification and qualification of NDEs using the Greyson NDE scale [8]. Participants whose experience did not meet the accepted cut-off score to be considered NDE experiencers- NDErs (i.e., Greyson total score ≥7; Greyson, 1983) were excluded from the study. Finally, the third part of the survey included an open-ended instruction (i.e., “Please use the space you need to narrate your NDE”) for the participants to retrospectively freely express their experience. Two specific requirements were set for the self-narrative: (a) written by the participant himself or herself and (b) described with respect to the context of occurrence and all perceptions. There was no space restriction for the written narrative. Like in any investigation dealing with retrospective self-reported phenomenological reports [17,18,25,26], the reliability of the narratives is challenging to determine both in terms of the participant’s good will and in the possible memory biases that can take place [27]. In addition to control for possible discrepancies, the investigators carefully looked into the completed questionnaire (including responses to demographical and context of occurrence questions as well as the scored items at the Greyson NDE scale [8]) and associated testimonial to account for reliability of the NDE narratives. After doing so, it seems rather unlikely that the participants’ testimonials were solely affected by confabulation or imagined phenomenology. In fact, our sample’s NDE accounts were highly structured and explicitly narrative with rich emotional components. Written completion of the anonymous survey was voluntary and taken as consent for participation in the study. The investigation was approved by the ethics committee of the Faculty of Medicine of the University of Liège.

**Fig 1:**
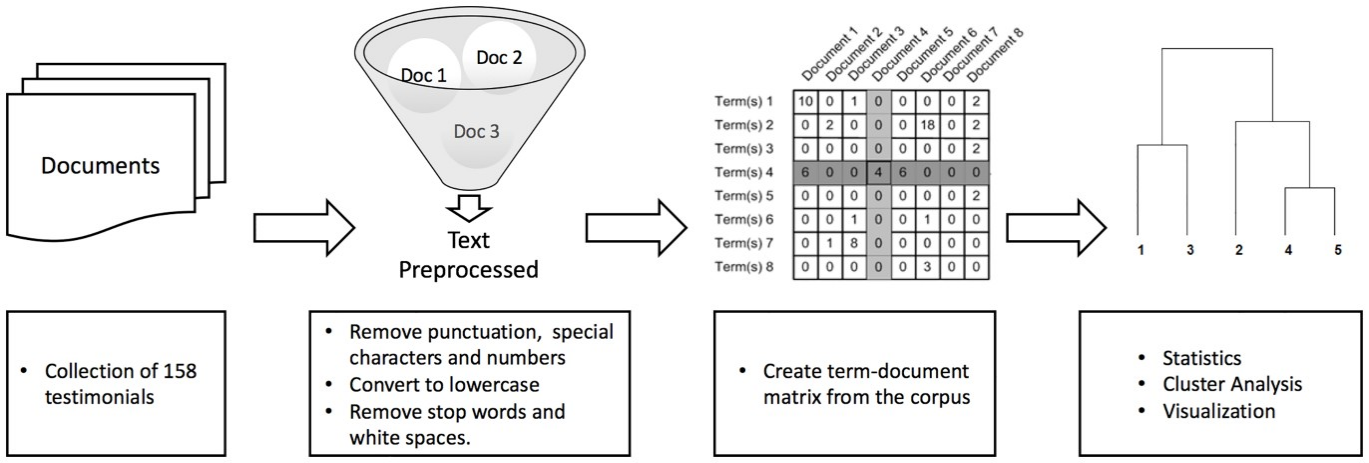
Overview of text classification procedure for characterization of NDE narratives.

The text mining analysis involved several steps, mainly influenced by the fact that texts, from a computer perspective, are rather unstructured collections of words. We first organized and structured the texts in a uniform manner. Once the texts were organized in a repository, the second step was tidying up the texts, including preprocessing the texts to obtain a convenient representation for the analysis. The third step consisted in transforming the preprocessed texts into structured formats by creating a so-called term-document matrix. Finally, standard techniques from statistics and data mining were applied including counting frequencies and subsequent clustering. Each one of the followed steps, as also schematically presented in Fig 1, are described below.

Data preprocessing was performed using R statistical computing language (version 3.4) using the text mining package tm (version 0.7–1; https://CRAN.R-project.org/package=tm [28] to: (a) remove punctuation and special characters; (b) remove numbers; (c) convert to lowercase; (d) remove the “stop words” without analytic value; (e) strip unnecessary white space from the documents; (f) remove words that didn’t bring any ‘‘meaning” to the analysis; and (g) group synonyms.

Before the analysis, we created the term-document matrix. Given a corpus of documents and a dictionary of terms containing all the words that appeared in the documents, the term-document matrix was obtained in a two-dimensional matrix form. Rows were filled with the terms (words) with the columns indicating the different documents, in such a way that each entry (i,j) represented the frequency of term i in document j [29–31]. Simple distance analysis was subsequently applied to the term-document matrix (see S2 Table).

Before performing hierarchical clustering, we calculated the similarity between each pair of words, which is referred to as the distance. We measured Euclidean distance to quantify dissimilarity between words. By assuming that each one of the words was a point in a n-space (with n the number of documents), the distance between two words (word X and Y) with coordinates (term frequencies) (*x*_1_,*x*_2_, …*x*_*n*_) and (*y*_1_,*y*_2_, …*y*_*n*_), was then calculated as the Euclidean distance between two points as given by:

$$D(X,Y)=\sqrt{{\left(x_{1}{-y}_{1}\right)}^{2}+{\left(x_{2}{-y}_{2}\right)}^{2}+\ldots +{\left(x_{n}{-y}_{n}\right)}^{2}}$$

The equation was used to create our distance matrix as also described in [32,33]. As reported in Maher *et al*., (2016), Euclidean distance is widely used in clustering problems, including clustering text and it is also the default distance measure used with the K-means algorithm [34]. See also [35] to compare Euclidean distance with other possible distance measures like Cosine and Jaccard when using K-means clustering technique. The distance matrix was subsequently used to create a dendrogram, by displaying a hierarchical relationship among the terms (words). Given a set of N items to be clustered, and an N*N distance (or similarity) matrix, we followed the basic process of hierarchical clustering as defined by Johnson et al. [31]. First, we started by assigning each item to be a cluster, so that if we had for example N items, we could initially assume that we had N clusters, each containing just one item. Second, we let the distances (similarities) between the clusters to be the same as the distances (similarities) between the items they contained. Third, we found the closest (most similar) pair of clusters and merged them into a single cluster, in order to have one cluster less. Forth, we computed distances (similarities) between the new cluster and each of the old clusters. Fifth, we repeated the second and third steps until all items were clustered into a single cluster of size N.

The hclust function, a complete linkage method, also called the diameter or maximum method as implemented in R, was used for our analysis in order to perform a hierarchical clustering. This clustering method defined the cluster distance between two clusters to be the maximum distance between their individual components [31,36]. This implied that at every stage of the clustering process, the two nearest clusters were merged into a new cluster. The process was repeated until the whole data set was agglomerated into one single cluster. The distance between two clusters was then finally calculated using the Lance-Williams update formula [37].

## Results

Table 1 shows the demographic characteristics of the study cohort recruited for their NDE retrospective narratives (N = 158; 83 females (53%); age at NDE 35±17y; time since NDE 21±14y). The NDE took place in various contexts life-threatening (i.e. with coma; n = 54; 34%) or not (i.e. no coma; n = 104; 66%). The medical causes with and without a period of coma included etiologies like anoxia (e.g., cardiac arrest, near-drowning), trauma (e.g. motor vehicle accident, falls) other medical causes (i.e., non-traumatic contexts such as an exacerbation of on-going illness, complication during surgery) and other non-medical causes (i.e. non-traumatic and non-life-threatening contexts such as during sleep or with no apparent cause).

**Table 1:** Sample’s demographics and NDEs’ contexts of occurrence (N = 158).

Demographics	NDE contexts			
	Anoxia (n = 53)	Trauma (n = 30)	Other medical cause (n = 64)	No medical cause (n = 11)
Gender—female	24 (45%)	14 (47%)	37 (58%)	8 (73%)
Age at NDE (mean in years ± SD)	43±17	25±13	31±15	41±11
Time since NDE	14±13	28±11	23±15	19±13

**Fig 2:**
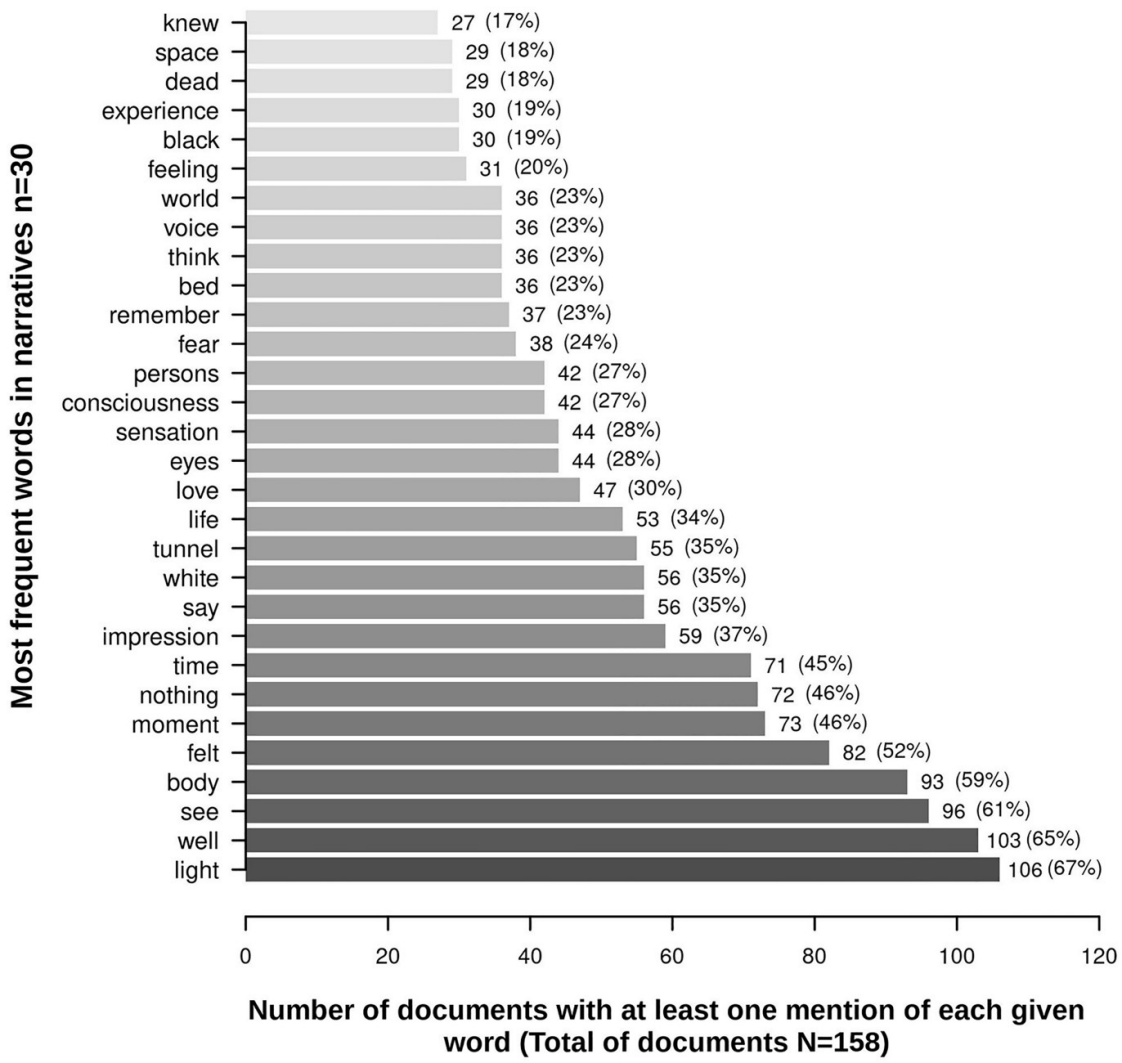
The 30 most frequent words/features in documents/experiencers’ narratives (N = 158).

If the individuals experienced more than one NDE, they were invited to choose their most important one. The mean length of the narratives was of 140 words per text (range 13–1592). The conducted analyses permitted us to extract 30 most reported features- features that were then used to draw a visual statistical map on their association. Fig 2 shows the 30 most reported features/words according to our analyses.

**Fig 3:**
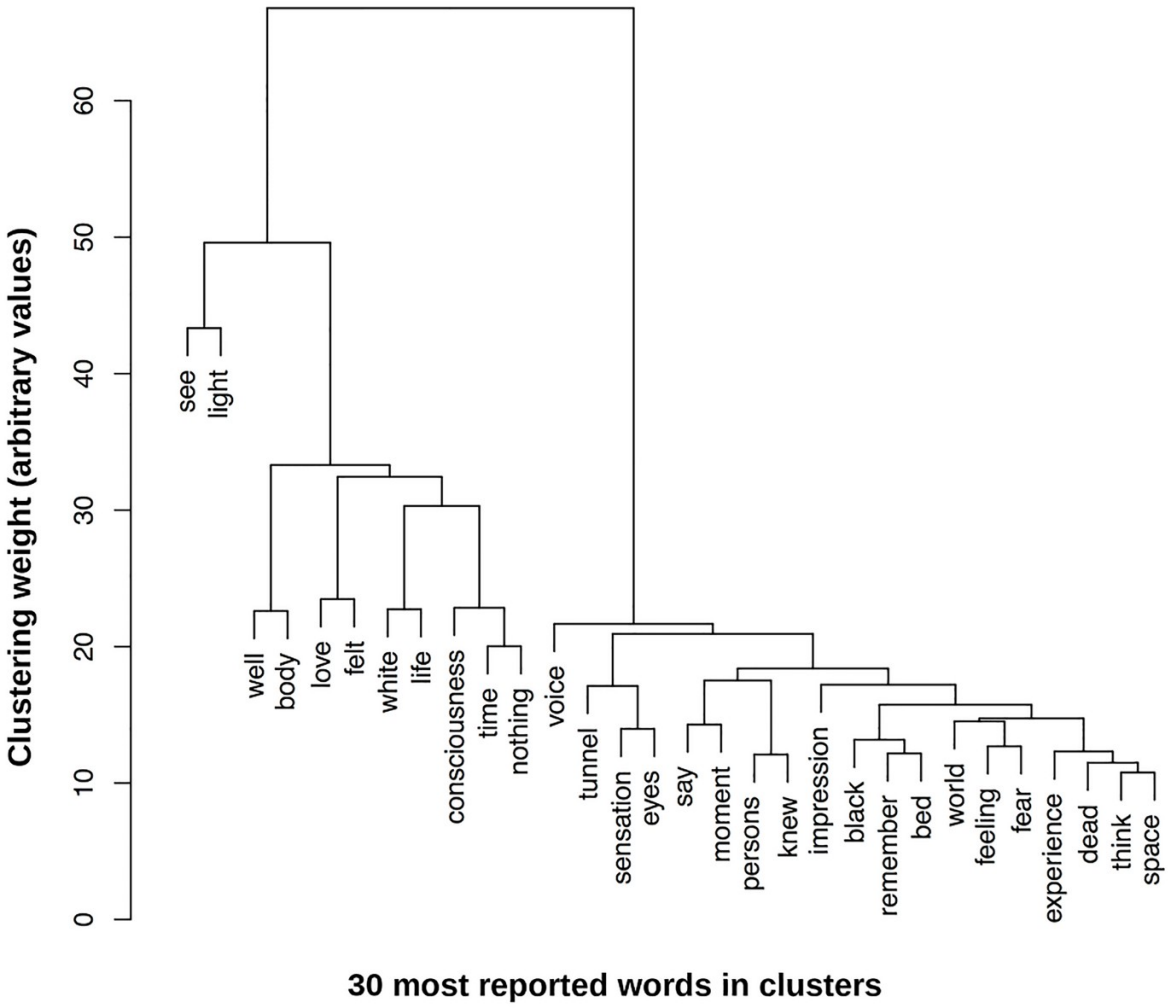
Dendrogram representing the hierarchical clustering of the most reported words (n = 30).

Fig 3 represents the clustering map (i.e., dendrogram) of these 30 most cited words in three main clusters. The dendrogram is a graphical output of the hierarchical clusters. Our analyses show that three main clusters stand out. The first cluster includes the words “see” and “light”. This cluster is closely correlated with the second cluster including words such as “well” and “body”, while the third and last cluster includes words such as “voice”, “tunnel”, “sensations and “eyes. Moving from left to right along the horizontal axis, we can observe that the words of the first cluster are separated from the others words to a decreasing degree. The scale presented by the vertical axis is the clustering weight, representing the distinction between the different clusters. In other words, “see” and “light” appear more often with “well” and “love” than with “dead” and “fear” among our sampled NDE testimonies. That is, visual perceptual features (e.g., “see”, “light”, “white”; first cluster) seem to be closer to positive emotions (e.g., “well”, “love”), than to spatial components (e.g., “tunnel”, “bed”, “space”), and also visual associated words (e.g., “tunnel”, “eyes”) of the second cluster. It can be observed that the emotional tone of the second cluster seems less positive (e.g., “black”, “fear”, “death”).

## Discussion

The first aim of this preliminary investigation using a text mining approach was to identify the main features (i.e., words elements) of freely expressed written NDEs. Fig 2 shows the 30 most reported words extracted from the 158 narratives. We can observe many similarities with previously identified Greyson NDE scale [8] features in the literature (i.e., feelings of peacefulness/well-being, out-of-body experience, seeing a bright light, an altered time perception [4,10–16]) with what was extracted in this study using text mining (i.e., see, light, body, time, voice). Moreover, our analyses revealed features that are consistent with the themes identified via thematic qualitative analyses [18] (i.e., light, darkness, tunnel, description of scenes, out-of-body experiences, awareness of death, time perception). In addition, the text mining analyses yielded extra features that are not part of the Greyson NDE scale such as tunnel (35%), love (30%), and fear (24%; Fig 2 & S2 Table). Some authors have argued that the tunnel perception in NDEs might be a culture-bounded phenomenon [38]. When comparing retrospective NDE accounts collected before and after Moody popularized NDEs in his best-seller [39], it was found that the tunnel feature was significantly more common in NDEs reported after 1975 than before. This suggests that the NDE model described by Moody might have influenced the perception of a tunnel [40]. In addition, some authors have argued that NDEs might not be universal in their content [38,40,41]. It would be interesting to use this technique to further investigate societal models and culture impacts on NDEs—considering how people from different backgrounds and time period describe their NDE. The second most frequent word/feature that our analyses highlighted was “love”. In fact, when interviewing NDErs, a substantial number of them will recall a feeling of unconditional love when they moved towards the light and/or when they encountered a mystical being or presence (e.g. [42–44]. Love seems to be a key feature in the experience and for its long-lasting emotional tone. In the NDE literature, the love feature is also discussed as an after-effect of the experience. It seems that for the majority of NDErs, the experience can lead to the capacity to express and feel unconditional love as well as a loss of materialistic values and fear of death [45]. According to the life-change inventory—a scale developed to assess self-image, concern with others, materialism and social issues, religious beliefs and spirituality, and attitude towards death [6,46]—it was shown that cardiac arrest patients with a NDE reported significantly more love and empathic attitude at a 2-year follow up [3]. The third most reported feature “fear” is interesting since it is not usually associated with a NDE [4,11,14]. To date, very few NDEs with negative emotions have been documented [6]. To date, it is estimated that negative or frightening NDEs account for about 1–2% of retrospectively collected reports [4,6]. This type of NDEs are possibly underestimated due to the individuals’ reluctance to disclose them since they might revive traumatic components [6]. Also of interest to mention that the Greyson NDE scale [8] does not permit the assessment of negatively connoted aspects of the experience, as already stated by Greyson and Bush (1992). It is also possible that NDEs contain both negative/frightening and positive/peaceful emotions in the same experience. For instance, some NDErs have previously reported having had transitory feelings of fright, which were replaced by tranquility as the experience unfolded [47]. Moreover, it was recently observed that the spatial location of the acquired brain damage that caused the coma or loss of consciousness triggering a NDE could have a potential effect on the emotional tone of the experience [5]. In fact, individuals with infratentorial lesion (i.e., lesions to the brainstem area and connections) would tend to report fewer positive features when questioned about their NDE using the Greyson NDE scale [2,8]. Although previous studies have suggested that experiencing a NDE during a life-threatening event might produce a positive effect on patients’ well-being and diminish psychological distress [43], a NDE filled with less positive and even negative emotions could potentially lead to the development of post-traumatic stress disorder (PTSD) and lead to a lower quality of life after the event. In these cases, some individuals would potentially benefit from a psychological follow-up including similar therapeutical goals to those of patients with PTSD [6,43], and as recent studies have shown that NDE memories are self-defining and constitute an important part of NDErs’ identities [48]. Surprisingly, although the NDEs’ popular association with religious concepts, our analyses failed to identify religious words. Previous work have identified religious features as a core concept and are included in the Greyson NDE scale (i.e. encountering mystical presences or deceased or religious spirits; items 14–15) [8,9]. The same authors also extracted transcendent or paranormal themes (e.g., angel, god, church, float, voice) in their quantitative semantic analysis paradigm. A recent study is Sri Lanka including hospitalized individuals showed that among those who reported a NDE, the patients of theistic religions (i.e. Christianity, Islam and Hinduism) reported more NDEs as compared with a non-theistic religious group (i.e. Buddhism) [49]. On the other hand, another research team in the Netherlands [3] failed to objectify an association between religious beliefs and occurrence of NDEs. The cultural background of the individuals experiencing a NDE is an important variable to account for and should be further studied using in particular quantitative automated and qualitative text analyses to enhance our understanding of the NDE phenomenon across the world.

The second aim of our investigation was to visualize the spatial correlation or statistical association’s strength (i.e., hierarchical clustering via the dendrogram; Fig 3) among the top reported NDE features. Hierarchical clustering has been widely used and is a popular tool in statistics and text mining approach for grouping data into “clusters”, leading to the identification of similarities or dissimilarities among narratives [28]. The dendrogram shows three main clusters of words. That is, visual perceptual features (e.g. see, light, white; first cluster) seem to be closer associated with positive emotions (e.g. well, “love), than to the spatial components (e.g. tunnel, bed, space), and also visual associate words (e.g. tunnel, eyes) of the second cluster. It can be observed that the emotional tone of the second cluster seems less positive (e.g. black, fear, death).

According to the spatial disposition, we can observe that the “positively toned” words stand far from the less positive ones. One could therefore speculate that, although the overall experience is reported as blissful, some parts of it might be less positive [18,50]. Like mentioned above, this finding is important since currently, the literature on negative NDEs remains a very poorly explored area.

Finally, some methodological limitations should be discussed. First, because the participants in this retrospective study were all individuals with a self-reported NDE and who, by the nature of this study’s form of data collection, were computer-literate, a potential source of selection bias might exist. The sample was likely not representative of the broader population with a NDE, favoring individuals with access to a computer and a certain level of writing ability. To address this potential issue, new applications of text-mining techniques—for instance, speech recognition where patients’ words can be automatically transferred into written forms—would bring extra benefits for both patients and investigators. With the use of speech recognition techniques, the potential NDErs would not have to write down their stories but could speak them out. Meanwhile, the data related to non-verbal communication, gestures and emotions could also be of importance in our goal to achieve a more comprehensive characterization of NDEs. Second, the retrospective recruitment of self-reported NDEs may not represent a reliable sample since the participants might have greater interest in and knowledge of NDEs. In addition to the probable sample-bias related to the recruitment of medically uncontrolled NDEs, the interval between the occurrence of the NDE and the age at study enrollment was several decades, similar to other retrospective surveys [10,15]. The retrospective design could also lead to memory biases although Greyson [51] found that memories of NDE seem to be consistent over time even after two decades. Therefore, as for other NDE retrospective studies investigating the experiencers’ subjective memory after many years, it should be stressed that our linguistic patterns results might reflect recalled perceptions rather than the core memory of their original NDE. Third, the user-independent automated approach described here does not permit to automatically return to the text to assess the meaning of a given word reported. For instance, the computer analysis does not distinguish between the “light” reported in the case of the NDE with the “light” that could be seen through a window or coming from a lighting in the surgical room. To address this issue, a next step would be to challenge our results with complementary qualitative experimenter dependent text analyses [16–18]. Finally, the choice of giving freedom into the written expression of NDEs gives rise to unstructured self-narratives that do not permit to control the amount of words and the construction of the narrative leading to discrepancies of the narratives’ content across subjects. It is probable that some information (e.g. emotions and/or particular perceptions) would better reveal their richness in a semi-directive data collection of NDE memories.

## Conclusions

Despite their circumstances of occurrence, NDEs are generally experienced as extremely pleasant and can induce life-changing consequences on the experiencers’ set of values and attitudes towards death [3]. In addition to the ill-described relation between the NDEs and the precipitating factors, the reliability of NDEs accounts remains controversial. The main finding of our preliminary study using a text mining approach is that there was a good agreement between the textual analyses and the literature [4,10–16]. Moreover, the text mining approach permitted to highlight a higher range of important features among the NDE narratives. We believe that the use of language processing via text mining techniques is a promising application to identify clinical and phenomenological information contained in unstructured freely expressed NDE testimonials [52]. For instance, automated speech analyses could further help in accurately discriminated between NDEs, NDEs-like (i.e., NDE phenomenology that is not associated with a life-threatening context; [4]). In fact, the technique could allow for group comparisons among etiology leading to the NDE but also to compare NDEs with other modified and dissociative states of consciousness that have been shown to include elements similar to a NDE (e.g. ketamine use [53], REM sleep [14,54], meditation [2], hypnosis [55], syncope [56]. The analyses could thus permit to test the hypotheses arguing that NDEs can be comparable to other altered states of consciousness [57]. In addition, our analyses permitted to extract “less positive” words (e.g.). It would be interesting as future work to use our material with sentiment analysis—a metric method that is commonly used to assess the intensity of positive and negative opinions and emotions in narratives (for an example see [58]). To add another dimension to our results, we could also use techniques related to word embeddings (e.g. word2vec) to better understand semantic relatedness and similarity between the words (for an example see [59]).

In conclusion, quantitative analysis and clustering techniques of text mining approach in this preliminary study provide the ability to visualize multivariate highly subjective raw material in 2D and thus give an additional point of view of the findings. Also, patterns that are not always obvious with raw eyes, sometimes also due to researchers’ objectivity, might be brought up. Additionally to the aforementioned, quantitative analysis and open source tools permit compatibility with future works by preserving the criteria/conditions of each research. Therefore, computerized analysis of complex human experiences via speech may present an opportunity to move our understanding of NDEs beyond reliance on self-report and questionnaire toward more objective measures of the phenomenon.

## Supporting information

S1 Table Detailed Greyson NDE scale. (PDF)

S2 Table Top 30 words statistics. (PDF)

S1 Code Annotated code to run the analysis in this paper. It also contains the lists of words with no meaning and used synonymous.
(PDF)
