# Supplementary material for: Characterization of near death experiences using text mining analyses: A preliminary study
Source: PLoS One. 2020 Jan 30;15(1):e0227402. doi: 10.1371/journal.pone.0227402 (PMC6992169; doi:10.1371/journal.pone.0227402)
Supplement: S1 Table — (PDF) [file pone.0227402.s001.pdf]

Table 1. Detailed Greyson NDE scale [5]

Questions/ features	Responses
1: Did time seem to speed up or slow down?	0 = No<br>1 = Time seemed to go faster or slower than usual<br>2 = Everything seemed to be happening at once; or time stopped or lost all meaning
2: Were your thoughts speeded up?	0 = No<br>1 = Faster than usual<br>2 = Incredibly faster
3: Did scenes from your past come back to you?	0 = No<br>1 = I remembered many past events<br>2 = My past flashed before me, out of my control
4: Did you suddenly seem to understand everything?	0 = No<br>1 = Everything about myself or others<br>2 = Everything about the universe
5: Did you have a feeling of peace or pleasantness?	0 = No<br>1 = Relief or calmness<br>2 = Incredible peace or pleasantness
6: Did you have a feeling of joy?	0 = No<br>1 = Happiness<br>2 = Incredible joy
7: Did you feel a sense of harmony or unity with the universe?	0 = No<br>1 = I felt no longer in conflict with nature<br>2 = I felt united or one with the world
8: Did you see, or feel surrounded by, a brilliant light?	0 = No<br>1 = An unusually bright light<br>2 = A light clearly of mystical or other-worldly origin
9: Were your senses more vivid than usual?	0 = No<br>1 = More vivid than usual<br>2 = Incredibly more vivid
10: Did you seem to be aware of things going on elsewhere, as if by extra sensorial perception/telepathy?	0 = No<br>1 = Yes, but the facts have not been checked out<br>2 = Yes, and the facts have been checked out

11: Did scenes from the future come to you?	0 = No<br>1 = Scenes from my personal future<br>2 = Scenes from the world’s future
12: Did you feel separated from your body?	0 = No<br>1 = I lost awareness of my body<br>2 = I clearly left my body and existed outside it
13: Did you seem to enter some other, unearthly world?	0 = No<br>1 = Some unfamiliar and strange place<br>2 = A clearly mystical or unearthly realm
14: Did you seem to encounter a mystical being or presence, or hear an unidentifiable voice?	0 = No<br>1 = I heard a voice I could not identify<br>2 = I encountered a definite being, or a voice clearly of mystical or unearthly origin
15: Did you see deceased or religious spirits?	0 = No<br>1 = I sensed their presence<br>2 = I actually saw them
16: Did you come to a border or point of no return?	0 = No<br>1 = I came to a definite conscious decision to “return” to life<br>2 = I came to a barrier that I was not permitted to cross; or was “sent back” against my will
