# Supplementary material for: Characterization of near death experiences using text mining analyses: A preliminary study
Source: PLoS One. 2020 Jan 30;15(1):e0227402. doi: 10.1371/journal.pone.0227402 (PMC6992169; doi:10.1371/journal.pone.0227402)
Supplement: S2 Table — (PDF) [file pone.0227402.s002.pdf]

Table 2. Top 30 words statistics

Top 30 Words	Total	Max	Mean	SD	In docs	% in docs
light	319	19	2.0	3.0	105	66
see	311	27	2.0	3.8	95	60
body	235	15	1.5	2.4	91	58
well	234	14	1.5	2.0	102	65
felt	175	8	1.1	1.6	81	51
nothing	160	12	1.0	2.0	72	46
time	147	10	0.9	1.7	70	44
life	135	14	0.9	1.8	53	34
love	130	15	0.8	2.0	47	30
consciousness	103	15	0.7	1.9	42	27
white	100	15	0.6	1.5	55	35
moment	99	6	0.6	1.0	63	40
impression	99	8	0.6	1.1	59	37
tunnel	94	9	0.6	1.2	55	35
say	87	5	0.6	1.0	56	35
sensation	75	5	0.5	1.0	44	28
voice	75	9	0.5	1.3	36	23
fear	71	9	0.5	1.1	38	24
persons	65	7	0.4	0.9	41	26
eyes	64	7	0.4	0.9	44	28
world	61	8	0.4	1.1	36	23
bed	57	5	0.4	0.9	36	23
think	57	6	0.4	0.9	35	22
experience	53	5	0.4	0.9	30	19
feeling	52	6	0.3	0.9	31	20
black	49	5	0.3	0.8	30	19
remember	47	4	0.3	0.7	37	23
space	47	4	0.3	0.7	29	18
knew	47	6	0.3	0.9	27	17
dead	46	7	0.29	0.8	29	18

Total: is the number of times each word was found among all the documents, **Max**: is the maximum number of times each word was found in just one of the documents, Mean: is the mean number of times the word was found among all the documents, **SD**: is the standard deviation for each word among all the documents, **in docs**: is the number of documents in which the word was found, **% in docs:** is the percentage of documents containing at least one time the word.
