# Supplementary material for: Characterization of near death experiences using text mining analyses: A preliminary study
Source: PLoS One. 2020 Jan 30;15(1):e0227402. doi: 10.1371/journal.pone.0227402 (PMC6992169; doi:10.1371/journal.pone.0227402)
Supplement: S1 Code — It also contains the lists of words with no meaning and used synonymous. (PDF) [file pone.0227402.s003.pdf]

```r
#################################################################################
# PLOS ONE - A better characterization of near death experiences using
#             text mining analyses: a preliminary study.
# This program is free software: you can redistribute it and/or modify
# it under the terms of the GNU General Public License as published by
# the Free Software Foundation - GPL V3
# This code is distributed in the hope that it will be useful,
# but without any warranty.
# Western University @uwo
# Copyright (C) 2018 Demetrius Ribeiro de Paula
#################################################################################

# Debian GNU/Linux 9 and R 3.4

# Load packages for text mining and then load the texts into R.
suppressPackageStartupMessages({
  library(tm) # Version: 0.7-1
  library(SnowballC) # Version:0.5.1
  library(ggplot2) # Version: 2.2.1
  library(cluster) # Version: 2.0.6
  library(FactoMineR) # Version: 1.39
  library(ape) # Version: 5.0
  library(dendextend) # Version: 1.7.0
 })
#### The documents are here:
folder_all <- "~/Nextcloud/University/Uwo/Text_Mining/NDE/Data/"
### Save figures and output here:
folder_save <- "~/Nextcloud/University/Uwo/Text_Mining/Plots_English/"
# Set the work folder
setwd(folder_all)
# Set Lowercase Function
tryTolower <- function(x) {
  y <- NA
  try_error <- tryCatch(tolower(x), error = function(e) e)
  if (!inherits(try_error, "error")) {
    y <- tolower(x)
  }
  return(y)
}
### Data Preparation
# Read and convert the documents to a data frame and then to a corpus.
docs <- Corpus(DirSource(folder_all))
toString <- content_transformer(function(x, from, to) gsub(from, to, x))
# The corpus transformations, including changing letters to lower case,
# removing punctuations/numbers and removing stop words.
docs <- tm_map(docs, content_transformer(tryTolower))
docs <- tm_map(docs, removeWords, stopwords("french"))
# Remove the words (Stop Words)
remove_words <- c(
  "apres", "avant", "avec", "chez", "concernant", "contre", "dans", "de", "depuis",
  "derriere", "des", "devant", "durant", "en", "entre", "envers", "hormishors",
  "jusque", "jusqua", "malgre", "moyennant", "nonobstant", "outre", "par", "parmi",
  "pendant", "pour", "pourtant", "pres", "sans", "sauf", "selon", "sous", "suivant",
```

```
  "sur", "touchant", "vers", "via", "jai", "jetais", "javais", "là", "je",
  "il", "tu", "nous", "vous", "elles", "ils", "comme", "tout", "puis", "tous", "toute",
  "toutes", "ans", "cetait", "cetait", "après", "derrière", "dès", "jusqu'à", "malgré",
  "près", "J'ai", "j'étais", "j'avais", "ça", "c'était", "jétais", "cétait", "jusquà",
  "jétais", "javais", "Jai", "autres", "droite", "dune", "dun", "quil", "nai", "car",
  "alors", "lorsque", "lorsqu'un", "lorsqun", "lorsqu'une", "lorsqune", "qu'une",
  "quune", "qu'un", "quun", "plus", "très", "cest", "c'est", "fait", "dit", "encore",
  "être", "quand", "deux", "aussi", "nest", "sest", "s'est", "autre", "avoir",
  "peux", "donc", "non", "quelque", "quelques", "l'on", "lon", "d'une", "dune",
  "c'était", "cétait", "vraiment", "veux", "suite", "aucune", "comment", "autour",
  "dis", "dit", "jours", "vais", "haut", "navais", "n'avais", "juste", "qu'on", "quon",
  "peut", "l'", "m'", "n'", "s'", "jen", "j'en", "parce", "que", "parce que", "ainsi",
  "peut-être", "l'ai", "lai", "nen", "n'en", "déjà", "sais", "deux", "trois", "quatre",
  "un", "dont", "trf", "tre", "eatre", "mais", "peu", "fois", "enfin", "elle", "passe",
  "semble", "etait", "etais", "coup", "une", "cote", "cette", "sens", "chose",
  "toujours", "jamais", "etat", "trop", "retrouve", "reste", "sorte", "beaucoup",
  "moins", "voulais", "instant", "demande", "maintenant", "tellement", "personne",
  "ensuite", "dois", "pouvais", "assez", "arrive", "train", "totalement", "retrouvee",
  "choses", "fond", "rester", "faire"
)
docs <- tm_map(docs, removeWords, remove_words)
docs <- tm_map(docs, stripWhitespace)
docs <- tm_map(docs, removeNumbers)
docs <- tm_map(docs, removePunctuation)
# Repalce the first word with the second
docs <- tm_map(docs, toString, " limpression", " impression ")
docs <- tm_map(docs, toString, " lamour ", " amour ")
docs <- tm_map(docs, toString, " damour ", " amour ")
docs <- tm_map(docs, toString, " lautre ", " autre ")
docs <- tm_map(docs, toString, "è", "e")
docs <- tm_map(docs, toString, "ê", "e")
docs <- tm_map(docs, toString, "é", "e")
docs <- tm_map(docs, toString, "ô", "o")
docs <- tm_map(docs, toString, " voir ", " vois ")
docs <- tm_map(docs, toString, " voyais ", " vois ")
docs <- tm_map(docs, toString, " vue ", " vois ")
docs <- tm_map(docs, toString, " senti ", " sentais ")
docs <- tm_map(docs, toString, " ressens ", " sentais ")
docs <- tm_map(docs, toString, " ressentais ", " sentais ")
docs <- tm_map(docs, toString, " blanc ", " blanche ")
docs <- tm_map(docs, toString, " gens ", " personnes ")
docs <- tm_map(docs, toString, " aucun ", " rien ")
# Remove stop words
docs <- tm_map(docs, removeWords, remove_words)
## Convert the top 30 words from French to English
docs <- tm_map(docs, toString, " lumiere ", " light ")
docs <- tm_map(docs, toString, " vois ", " see ")
docs <- tm_map(docs, toString, " bien ", " well ")
docs <- tm_map(docs, toString, " corps ", " body ")
docs <- tm_map(docs, toString, " sentais ", " felt ")
docs <- tm_map(docs, toString, " rien ", " nothing ")
docs <- tm_map(docs, toString, " temps ", " time ")
docs <- tm_map(docs, toString, " vie ", " life ")
```

```
docs <- tm_map(docs, toString, " amour ", " love ")
docs <- tm_map(docs, toString, " conscience ", " consciousness ")
docs <- tm_map(docs, toString, " impression ", " impression ")
docs <- tm_map(docs, toString, " moment ", " moment ")
docs <- tm_map(docs, toString, " tunnel ", " tunnel ")
docs <- tm_map(docs, toString, " dire ", " say ")
docs <- tm_map(docs, toString, " blanche ", " white ")
docs <- tm_map(docs, toString, " sensation ", " sensation ")
docs <- tm_map(docs, toString, " voix ", " voice ")
docs <- tm_map(docs, toString, " peur ", " fear ")
docs <- tm_map(docs, toString, " yeux ", " eyes ")
docs <- tm_map(docs, toString, " monde ", " world ")
docs <- tm_map(docs, toString, " lit ", " bed ")
docs <- tm_map(docs, toString, " pense ", " think ")
docs <- tm_map(docs, toString, " personnes ", " persons ")
docs <- tm_map(docs, toString, " experience ", " experience ")
docs <- tm_map(docs, toString, " noir ", " black ")
docs <- tm_map(docs, toString, " sentiment ", " feeling ")
docs <- tm_map(docs, toString, " espace ", " space ")
docs <- tm_map(docs, toString, "mort ", " dead ")
docs <- tm_map(docs, toString, "savais ", " knew ")
docs <- tm_map(docs, toString, " souviens ", " remember ")
# Create a matrix with terms as rows and documents as columns
tdm <- TermDocumentMatrix(docs)
m <- as.matrix(tdm)
# Calculate the words frequency
palavras_freqs <- sort(rowSums(m), decreasing = TRUE)
dm <- data.frame(word = names(palavras_freqs), freq = palavras_freqs)
dm$Perc <- dm$freq / sum(dm$freq) * 100
percentage <- format(head(as.matrix(dm$Perc), 30), digits = 2, nsmall = 1)
top_palavras <- head(palavras_freqs, 30)
bigvaluew <- as.matrix(top_palavras)
bigvaluew <- bigvaluew[1] + bigvaluew[1] / 8
fatorw <- bigvaluew * 3 / 100
number_of_words <- sum(dm$freq)
# Top 30 All words
top_words <- data.frame(words = top_palavras) # All top 30 words
# Frequency of each word by document
matrix <- merge(
  y = m, x = top_words, by.y = "row.names", by.x = "row.names",
  all.x = TRUE
)
rownames(matrix) <- matrix[, 1]
matrix[, c(1)] <- NULL
# More stats about the words across the documents
top_words_stats <- data.frame(
  in_docs = rowSums(matrix != 0), Sum = rowSums(matrix[, -1]),
  Min = apply(matrix[, -1], 1, function(x) min(x)),
  Max = apply(matrix[, -1], 1, function(x) max(x)),
  Mean = rowMeans(matrix[, -1]),
  SD = apply(matrix[, -1], 1, sd, na.rm = TRUE)
)
top_words_stats$percentage_in_docs <- (top_words_stats$in_docs / length(docs)) * 100
```

```
top_words_stats$words <- rownames(top_words_stats)
top_words_stats <- top_words_stats[, c(8, 1, 2, 3, 4, 5, 6, 7)]

### Table 2 - Top 30 All words (Stats)
top_words_stats <- top_words_stats[order(-top_words_stats$in_docs), ]

### Plot Figure 2
percentage <- format(round(sort(top_words_stats$percentage_in_docs, decreasing = TRUE)),
  digits = 0, nsmall = 0
)
top_palavras2 <- top_words_stats[, -c(1, 3, 4, 5, 6, 7, 8)]
bigvaluew <- as.matrix(top_palavras2)
bigvaluew <- bigvaluew[1] + bigvaluew[1] / 8
fatorw <- bigvaluew * 3 / 100
title <- paste("Top_30_Words_NDE # of words = ", number_of_words, sep = "")
filename <- paste(folder_save, "Gray_Top_Words_Among_Docs_NDE.tiff", sep = "")
tiff(
  file = filename, width = 2000, height = 2000, units = "px", res = 400,
  compression = "lzw", pointsize = 7
)
bplt <- par(mar = c(4, 7.2, 3, 2))
bplt <- barplot(top_palavras2,
  border = NA, las = 1, main = title, xlab = "# of unique times among the documents",
  cex.main = 1.4, cex.lab = 1.4, cex.sub = 1.2,
  horiz = TRUE, cex.names = 1.1, axis.lty = 1, col = gray.colors(30),
  xlim = c(0, bigvaluew + 5), names = top_words_stats$words
)
text(
  x = top_palavras2 + fatorw, y = bplt, labels = as.character(top_palavras2),
  xpd = TRUE, cex = 0.95
)
text(
  x = top_palavras2 + 2.95 * fatorw, y = bplt,
  labels = as.character(paste0("(", percentage, "%", ")")), xpd = TRUE, cex = 0.95
)
invisible(dev.off())

### Plot Figure 3
filename <- paste(folder_save, "Dendograma.tiff", sep = "")
tiff(
  file = filename, width = 2000, height = 2000, units = "px", res = 400,
  compression = "lzw", pointsize = 7
)
dend <- matrix[, -1] %>%
  dist(method = "euclidean") %>%
  hclust(method = "ward.D2") %>%
  as.dendrogram() %>%
  hang.dendrogram(hang_height = 2) %>%
  sort(type = "nodes") %>%
  set("branches_lwd", 1.2) %>%
  plot(main = "All Subjects")
invisible(dev.off())
###
```
